# The relationship between epicardial fat tissue thickness and transit time flow measurement values of coronary artery bypass grafts

**DOI:** 10.34172/jcvtr.2020.50

**Published:** 2020-11-24

**Authors:** Hacı Ali Uçak

**Affiliations:** ^1^Department of Cardiovascular Surgery, University of Health Sciences Adana City Training and Research Hospital, Adana, Turkey

**Keywords:** Epicardial Fat Tissue, Coronary Artery Bypass Grafting Surgery, Transit Time Flow Measurement

## Abstract

***Introduction:*** Epicardial fat tissue, the true visceral adipose depot of the heart, has been associated with changes in both cardiac function and morphology. This study aimed to show the relationship between epicardial fat tissue (EFT) thickness and graft flow dynamics in arterial and venous grafts in coronary artery bypass graft surgery (CABG).

***Methods:*** Patients underwent transthoracic echocardiography before surgery and epicardial fat thickness were evaluated. The patients were divided into two groups as EFT value <5.5 (group 1) mm and ≥5.5 (group 2) mm. One hundred eighty-one patients with a total of 434 grafts (162 arterial and272 venous) underwent isolated coronary artery bypass grafting surgery. All grafts were examined by transit time flow meter intraoperatively.

***Results:*** The mean epicardial fat tissue thickness values were 4.9±0.8 mm and 6.1±1.3 mm, respectively.Mean graft flow values of left internal mammary artery was 44.21±23.2 mL/min in group 1 and39.65 ± 19.2 mL/min in group 2 (*P *= 0.041). Similarly, mean graft flow values were higher in group1 in all venous grafts regardless of which vessel bypass was performed. There is a significant negative correlation between epicardial fat thickness and mean graft flow.

***Conclusion:*** Epicardial fat thickness measurement preoperatively might provide additional data for the faith of the graft.

## Introduction

The association between epicardial fat tissue (EFT) and cardiovascular diseases (CVDs) have been reported in previous studies.^1,2^ The EFT is thought of as an independent factor in myocardial modulation and development of atherosclerosis coronary arteries, but it is not a direct functional part of the heart.^3^ Today, in clinical practice, EFT thickness is a parameter that can be measured using standard 2D echocardiography efficiently. EFT, especially around subepicardial coronary arteries, is thought to lead perivascular inflammation and pathologic proliferation of smooth muscle cell. EFT has been reported to cause endothelial dysfunction and as a result development of early restenosis after coronary revascularization.^4^ Coronary artery bypass graft surgery (CABG) is a crucial revascularization method with proven success. The effectiveness of coronary surgery is also directly related to the natural vessel flow dynamics of the anastomosis. Previous studies have reported that intra-operative evaluation of graft function was performed effectively with Transit time flow measurement (TTFM).^5,6^ Albeit the relationship between EFT with atherosclerotic vascular damage coronary artery is prominent. To investigate the relationship between EFT thickness and transit time flow quantifications of venous and arterial grafts of coronary arteries in diabetic patients is the primary purpose of the current study.

## Materials and Methods

**Figure 1:**
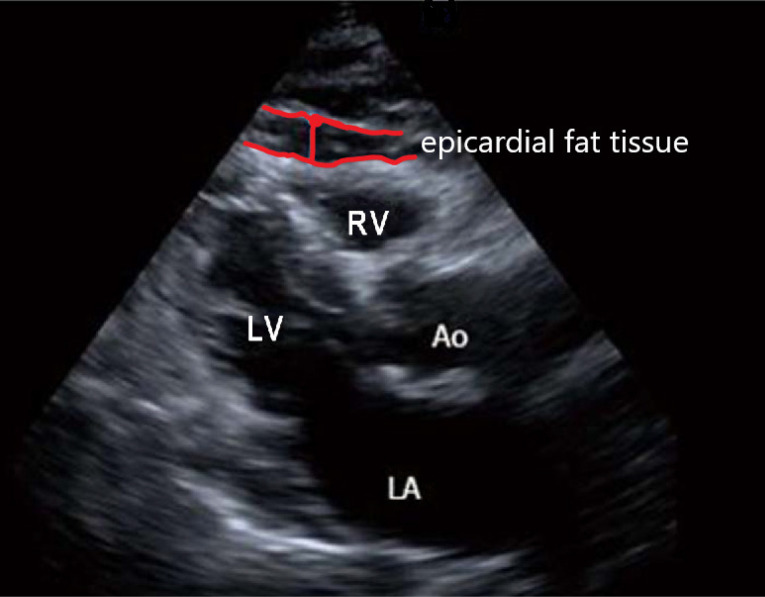
An exemplary view of epicardial adipose tissue thickness measurement

**Figure 2:**
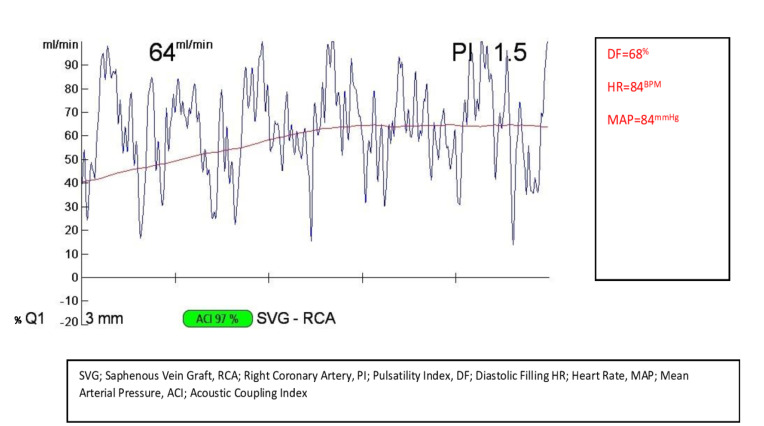
A sample of TTFM measurement report of saphenous vein graft anastomosis to the right coronary artery

A total of 181 subjects with stable coronary artery disease (CAD), who underwent CABG surgery under elective circumstances were registered to our retrospective study. Cukurova University ethical committee approved the study (meeting 91, decision number 56). The patient suffers from cardiac failure (systolic ejection fraction lower than 40%), body mass index (BMI) bigger than 40 kg/m^2^, and the patients we could not obtain an optimal echocardiographic measurement were excluded. Patients underwent coronary angiography, transthoracic echocardiography before elective surgery routinely. Demographic data, including age, gender, weight, height, BMI was collected. Also, if the patients had diabetes, hypertension, or smoking, their medical history records were registered (Table 1). A cardiologist performed the echocardiographic examination. The standard equipment of our clinic (Vivid-7, GE Vingmed Sound, Horten, Norway) was used for standard 2D and Doppler echocardiographic examination of patients preoperatively with a 2.5-3.5 MHz transducer. EFT was defined as a zone between the epicardial and the pericardial visceral layer. EFT was evaluated as a track opposite to the lateral free portion of the right ventricle at the standard parasternal long axial see in the left parallel position (Figure 1). A mean value was obtained after the maximum levels were measured at the end-diastolic phase of 3 serial cardiac cycles. Operation protocol All patients underwent CABG surgery in elective circumstances under general anaesthesia, and with cardiopulmonary bypass (CPB). According to routine clinical practice, anaesthesia management was conducted. For induction of anaesthesia, 0.1-0.2 mg/kg intravenous midazolam, 5-10 μg/kg fentanyl and 0.08-0.10 mg/kg vecuronium were administered to patients. Sevoflurane inhalation sustained the depth of anaesthesia. 320-400 IU/kg unfractionated heparin was administered by anaesthesiologists to maintain an activated clotting time (ACT) > 480 seconds. Ascending aorta was cannulated and then a single two-stage atrial cannula or bicaval cannulation used for venous drainage. 1350 mil prime volumes (1 L isolate S, 20% mannitol 200 cc, 3% NaCl, 150 cc, 5000 İU heparin) were admitted. 2.0 – 2.5 L/min/m² flow rate, 180 –200 mm Hg Pao2 and 35-45 mm Hg PCO_2_ was maintained. Warmblood cardioplegia was used for myocardial protection. A membrane oxygenator and an arterial line filter used for CPB, during CPB for all patients, aimed at the level of host > 22 %, mean arterial pressure was preserved at 60-80 mm Hg, serum glucose was kept between 110- 180 mg/dL with an infusion of insulin, cooled at 33-34 °C (nasopharyngeal core body temperature) and a-stat pH management as considered. Calculating the ultrasound waves passing from the transducer to the receiver is the basis of the TTFM measurement method: two Doppler signals are emitted and received in the opposite direction by two quartz crystals set at 45° angle with the bloodstream. The coronary graft which wants to be evaluated is placed into a flow probe in the middle of the two ultrasonic transducers and a reflector, in a vertical style. The transit time is the delayed time between transducer reflector and the receiver.^7^ The flow volume in the coronary graft is calculated by the flow meter based on the supplied transit time.^8^ Measurements of flow parameters of the grafts were performed after the cardiopulmonary bypass was removed and hemodynamically stabilization was completely achieved. Graft flow parameters were measured by utilizing a Transit-time flow meter (TTFM) (VeriQ System, Medistim, Oslo, Norway) before the skin closure. The mean graft flow, diastolic filling, pulsatility index, and systolic reverse flow were measured for each graft. (Figure 2) The diastolic filling value should be higher than 25% to be considered as a satisfactory graft.^9,10^ The best parameter of TTFM for the good anastomotic flow pattern might be the pulsatility index. This rate is obtained by dividing the difference between the maximum graft flow and the minimum graft flow by the mean flow:

**Table 1:** Demographic data

**Variable**	**Group 1** **(EFT thickness <5.5 mm)** **n=116**	**Group 2** **(EFT thickness ≥ 5.5 mm)** **n=65**	***P*** ** value**
Male, n (%)	67(57.7)	39(60)	0.195
Age, mean ± SD, y	66.8± 6.1	64.2 ± 6.9	0.163
Smoking, n (%)	27(23.2)	18 (27.6)	0.051
Hypertension, n (%)	24(20.6)	23(35,3)	< 0.001
Dyslipidemia, n (%)	25(21.5)	22(33.8)	0.078
Body Mass Index mean ± SD, kg/m^2^	33.4±5.8	32.1±6.7	0.023
Body surface area, m^2^	1.8±0.39	1.8±0.25	0.711
Total cholesterol, mg/dL	192.4±27.9	217.4±38.7	0.021
Low-density lipoprotein, mg/dL	111.0±19.2	131.0±30.0	0.634
Triglyceride, mg/dL	141.1±45.1	168.1±39.2	0.012
Creatinine, mg/dL	0.78±0.28	0.83±0.18	0.062
Haemoglobin, g/dL	13.9±2.1	13.6±1.8	0.105
Haemoglobin A1c, (%)	5.68±1.8	7.80±2.8	<0.001
Hs-CRP, mg/dL	0.62 ± 0.5	0.91 ± 0.6	0.041
Systolic blood pressure, mm Hg	130.5±12.1	135.1±17.8	0.365
Diastolic blood pressure, mm Hg	82.4±10.4	79.6±11.8	0.205
Heart rate, beats/min	75.3±14.6	78.7±13.1	0.094
History of cerebrovascular event, n (%)	3 (2.5)	7 (10.7)	<0.001
Left ventricular ejection fraction, (%)	64.30 ± 8.1	59.88± 7.3	0.048
Epicardial Fat Thickness ,mean ± SD, mm	4.9± 0.8	6.1± 1.3	<0.001

Abbreviations: Hs-CRP, high-sensitivity C-reactive protein

Pulsatility index = (maximum graft flow – minimum graft flow / mean graft flow).

For a successful anastomosis of a graft pulsatility index should range from 1 to 5; an index < 3.0 is a satisfactory value for a successful anastomosis.^11^ Systolic backward flow predicts a competitive status between the coronary artery and arterial or venous coronary graft in the course of the systolic period of heart beating cycles. The backward flow values greater than ≥3. 0% should be considered as a predictor parameter for early graft failure.

### Statistical analysis

SPSS 25.0 version (SPSS Inc., Chicago, Illinois) was utilized to analyse our data. Data are expressed as mean ± S and *P* < 0.05 is considered significant. Quantitative variables analysed by using the t-test, and the Mann-Whitney test was preferred to use if these variables were not normal. Pearson’s chi-squared test was used to analyse qualitative variables, and Fisher’s exact test was utilized when it is necessary. Multiple logistic regression model was utilized to evaluate the variables at the same time.

## Results

434 grafts in 181 patients who underwent elective CABG surgery under general anaesthesia with CPB were evaluated within the study. All the grafts were measured by TTFM intraoperative, 162 grafts were arterial, and 272 were venous (Table 2). Higher frequency of hypertension (35.3 % vs 20.6%,*P* < 0.001), history of the cerebrovascular event (10.7% vs 2.5% *P* ≤ 0.001) and the higher level of dyslipidaemia (33.8% vs 21.5%, *P* = 0.078), total cholesterol (217.4±38.7 mg/dL vs 192.4±27.9 mg/dL, *P* = 0.021), triglyceride (168.1±39.2 mg/dL vs 141.1±45.1 mg/dL, *P* = 0.012), creatinine (0.83±0.18 mg/dL vs 0.78±0.28 mg/dL, *P* = 0.062), haemoglobin A1C, CRP and lower levels of haemoglobin were observed in group 2 when compared with patients in group 1 . The mean EFT thickness were 4.9 ± 0.8 mm and 6.1 ± 1.3 mm respectively. EFT thickness was strongly correlated with older age (r =0.290, *P* < 0.001), dyslipidaemia (r = 0.512, *P* < 0.001), BMI (r = 0.512, *P* < 0.001) in bivariate analysis. EFT thickness had a negative correlation with the mean graft flow (r = -0.576, *P* < 0.001) and pulsatility index (r = 0.669, *P* < 0.001), however, a significant correlation was not found between EFT thickness and the diastolic filling (r = -0.238, *P* = 0.534) and reverse flow (r = 0.038, *P* = 0.805) values. There was a correlation between mean graft flow and age (r = 0.380, *P* < 0.001), dyslipidaemia (r = 0.684, *P* < 0.001), BMI (r = 0.690, *P* = 0.006), hypertension (r=0.420, *P* = 0.041), epicardial fat thickness (r= 0.915, *P* = 0.041) and DM (r=1.214, *P* = 0.018) in bivariate analysis. Univariate relationships and multivariable analyses of mean graft flow presented in Table 3.

**Table 2:** Number, mean graft ﬂow, diastolic filling, and pulsatile index and reverse flow values

**Group 1**	**n=116**	**Mean graft flow**	**Diastolic filling**	**Pulsatility index**	**Reverse flow**
Lima -Lad	109	44.21± 23.2	71 ± 17.5	2.2 ± 1.38	1.3 ± 0.63
Svg-Lad	7	48.52 ± 13.6	68 ± 16.8	1.7 ± 1.09	2.0 ± 0.76
Svg- Diagonal Branches of Lad	37	40.61 ± 14.8	62 ± 19.9	1.9 ± 1.13	1.6 ± 0.52
Svg-Circumflex Coronary Artery	65	39.30 ± 15.4	61 ± 17.1	1.7 ± 1.07	1.8 ± 1.62
Svg-Rca	69	53.62 ± 19.1	63 ± 25.3	1.6 ± 0.92	2.1 ± 1.27
**Group 2**	**n=65**	**Mean graft flow**	**Diastolic filling**	**Pulsatility index**	**Reverse flow**
Lima -Lad	53	39.65 ± 19.2	62 ± 14.2	2.6 ± 2.20	1.9 ± 0.48
Svg-Lad	12	44.34 ± 17.8	59 ± 17.3	2.1 ± 1.23	2.7 ± 0.54
Svg-Diagonal Branches of Lad	21	38.71 ± 15.8	60 ± 20.9	1.9 ± 1.35	2.5 ± 1.17
Svg-Circumflex Coronary Artery	32	36.40 ± 16.9	55 ± 13.6	2.8 ± 2.01	2.6 ± 1.09
Svg-Rca	29	47.14 ± 21.3	59 ± 24.4	2.2 ± 1.71	2.4 ± 1.43

Abbreviations: Lima, left internal mammary artery; Lad, left anterior descending artery; Svg, saphenous vein graft; Rca, right coronary artery

**Table 3:** Univariate relationships and multivariable analyses of mean graft flow

**Values**	**Pearson correlation coefficients**	***P***	**Standardized regression coefficients**	***P***
Age	0.380	<0.001	0.052	0.742
Hypertension	0.420	0.041	0.854	0.078
Dyslipidemia	0.684	<0.001	0.254	0.023
Hs-CRP	0.558	0.089	0.086	0.010
BMI	0.690	0.006	0.112	<0.001
DM	1.214	0.018	1.325	0.002
Epicardial Fat Thickness	0.915	0.041	0.214	0.035

Abbreviations: BMI, body mass index; DM, diabetes mellitus

## Discussion

To evaluate the predictive role of EFT in graft flow dynamics is the primary purpose of this study. İn some recent studies EFT has been reported to be a novel predictive sign of metabolic disorders and cardiovascular diseases independently.^11,12^ Previous studies reported that epicardial adipose tissue is a participating factor in the pathogenesis of CAD and there is a meaningful correlation between EFT and the severity of CAD, as strong as satisfactory.^13,14^ EFT might suppress İnsulin sensitivity and adiponectin secretion. İn adipose tissue, these factors induce inflammation, trigger the secretion process of proinflammatory cytokines also contribute to the development of type 2 diabetes mellitus. Relationship of these metabolic disorders and progression of coronary microvascular disorders is still not clear. Previous studies have shown that higher adipose tissue volume are thought to induce coronary microvascular dysfunction.^12^ Endothelial dysfunction as a cause of increased microvascular resistance is an early finding before clinically stenosis develops in patients with cardiovascular risk factors.^15^ Many studies have shown increased EFT volume in coronary artery stenosis.^16,17^ İn the present study, according to our results, poor TTFM values were observed in patients with higher EFT volumes. For a successful coronary arterial revascularization, in CABG surgery, it is mandatory that the graft flow passing through the anastomosis need to overrun the coronary flow. Several factors (graft diameter, plaque morphology of the coronary vessel, also type and quality of the grafts, the collateral flow from inside the native coronary vessel as well as the operational success of the anastomosis) affect the potency of a coronary artery bypass. Only some of these parameters depend on the surgery. Especially distal coronary resistance and collateral flow is hard to estimate before surgery. Akasaka et al showed that diabetic patients have a lower coronary flow reserve compared with nondiabetic individuals, but the underlying mechanism is still unclear.^18^ TTFM has become a popular method to evaluate the revascularization quality during CABG operations. Because, TTFM is non-invasive, easy to perform, rapid, simple to reproducible, and a characteristic sample of the real flow in the graft. İt is well known that, mean graft flow, diastolic filling, pulsatility index and reverse flow parameters previously proposed as predictors a higher prevalence of graft failure in the early period of surgery, for both arterial and venous conduits.^19^ Mean graft flow is a useful indicator for how graft is flowing after anastomosis and is expressed in ml/minute. However, it is not a solid marker of the classification of the anastomosis on the grounds that the index is affected by numerous factors, such as coronary microvascular resistance blood pressure and harvesting quality of the grafts. Many studies concerning interpretive coronary graft flow measurement have been published; greater than 20 mL/minute mean graft flow is defined as a satisfactory result. The diastolic filling is expressed as a percentage and signifies the ratio of diastolic flow during a total period of graft flow. The diastolic filling should range from 45% to 80% for an appropriate graft. ESC/EACTS guidelines on myocardial revascularization published in 2010 endorsed TTFM low mean graft flow lower than 20 mL/min and high pulsatility index greater than 5 to predict technically inadequate grafts, advocating graft revision before leaving the operation room.^20^ The power of TTFM to predict the potency of grafts in the long term is still controversial. A recent review study performed by Niclauss revealed the advantages and some shortcomings of the routine use of TTFM measurements. As a result of 9 studies screened, the graft failure rate was 12% and they concluded that “TTFM alone may not be a sufficient evaluation method to detect graft failure and other parameters should be specified. With the TTFM method, the sensitivity of the graft evaluation (ie the true positive rate) varies between 94% and 98%, but the specificity (ie true negative rate) rate is quite low, such as 61%.^21^ In a study completed by Tokuda and et al, In patients who underwent angiography follow-up for a mean of 16.5 ± 7.6 months’ lower mean graft flow (OR: 0.96, 95% CI: 0.93–0.98; *P* < 0.01) and a higher % diastolic filling (OR: 1.08, 95% CI: 1.01–1.17; *P* < 0.05) the measurements were inversely proportional to the graft opening in the mid-range.^19^ In the present study, we did not detect any meaningful relationship between EFT and reversed flow. Already we have not identified any graft competition, according to our clinical observations. Dynamic variables such as blood pressure, graft diameter, flow competition, heart rate and distal coronary resistance are some restrictive factors for optimal evaluation by affecting the graft assessment by TTFM. Saad F. Jaber et al reported that mean graft flow did not decrease significantly until graft stenosis was greater than 75 %. ^22^ There are some limitations in some points of our study. In this retrospective study, it was not possible to detect territory that had lost the viability due to previous infarctions. It is also obvious that tissue that has lost its vitality in the areas fed by the anastomoses will potentially have a negative effect on the graft flow. However, none of the patients included in our study underwent a surgical procedure requiring resection due to large myocardial scar tissue. On the other hand, with viability studies that are not routinely applied to every patient, measurements of graft flows can be performed more accurately. EFT might provide additional information and be a useful guide for vascular endothelial dysfunction and the faith of grafts. In this study, we performed EFT thickness measurements by echocardiography. Although echocardiography is fast, practical and easy to use, subjective results may emerge in part because it is dependent on the experience of the user. High-speed computed tomography and magnetic resonance imaging may give more accurate results, but it will not be efficient to use it for EFT thickness measurement since it is both expensive and impractical. Moreover, there are technical differences in how to evaluate tissue thickness with echocardiography among unlike studies, including measuring thickness in cardiac systole or diastole, used echocardiographic window type, and a location in each window that thickness is measured.

## Acknowledgments

The author gives special thanks to the staff of the department of cardiology of the University of Health Sciences Adana City Training and Research Hospital for their collaboration in echocardiographic assessment.

## Conclusion

EFT thickness might be associated with the mean graft flow, pulsatility index and diastolic filling. There is a stronger relationship between EFT thickness with the mean graft flow, pulsatility index and diastolic filling.

## Competing interests

No conflict of interest was declared by the authors.

## Ethical approval

Çukurova University ethical committee approved the study (meeting 91, decision number 56).

## Funding

The authors declared that this case has received no financial support.
